# The Influence of Facial Characteristics on the Relation between Male 2D:4D and Dominance

**DOI:** 10.1371/journal.pone.0143307

**Published:** 2015-11-23

**Authors:** Jan Ryckmans, Kobe Millet, Luk Warlop

**Affiliations:** 1 Faculty of Economics and Business, Research Center for Marketing and Consumer Science, KU Leuven, Leuven, Belgium; 2 Department of Marketing, VU University Amsterdam, Amsterdam, Netherlands; 3 Department of Marketing, BI Norwegian Business School, Oslo, Norway; State University of New York at Oneonta, UNITED STATES

## Abstract

Although relations between 2D:4D and dominance rank in both baboons and rhesus macaques have been observed, evidence in humans is mixed. Whereas behavioral patterns in humans have been discovered that are consistent with these animal findings, the evidence for a relation between dominance and 2D:4D is weak or inconsistent. The present study provides experimental evidence that male 2D:4D is related to dominance after (fictitious) male-male interaction when the other man has a dominant, but not a submissive or neutral face. This finding provides evidence that the relationship between 2D:4D and dominance emerges in particular, predictable situations and that merely dominant facial characteristics of another person are enough to activate supposed relationships between 2D:4D and dominance.

## Introduction

Animal and human studies suggest that prenatal exposure to testosterone influences both brain development and behavior [1]. As it is difficult (in animals) or even impossible (in humans) to directly measure prenatal exposure to testosterone levels, the second to fourth digit ratio (shortly 2D:4D) has increasingly been adopted as an indirect biomarker for prenatal testosterone exposure. Much evidence suggests that lower 2D:4D indicates a higher level of prenatal testosterone exposure [2, 3, 4, 5]. During the last decade abundant evidence has accumulated for the profound impact of prenatal testosterone exposure, indexed by 2D:4D, on personality traits, e.g. Sensation Seeking [6], trait physical aggression [7], implicit power motivation [8] and dominance [9], as well as on actual behavior, e.g. responses to infidelity threat [10], athletic prowess [11], and agreeableness towards women [12]. In the present paper we focus on the relationship between 2D:4D and dominance.

In biology, the capacity of 2D:4D to serve as a biomarker of dominance is well documented. The relationship between 2D:4D and dominance has been found at the species level: the competitive chimpanzee has a lower 2D:4D than the less competitive bonobo [13], and generally there is evidence that species with more competitive social systems have lower 2D:4D ratios [14]. Within species, low 2D:4D is associated with higher dominance rank in primates such as rhesus macaques [15] and baboons [16, 17].

In human research, the evidence for a similar relationship is mostly circumstantial. Several findings suggest that low 2D:4D reveals the need to achieve, to win, or to outdo others [18]. Indeed, low 2D:4D is related to success in sports such as professional football, rugby, rowing, endurance running, slalom skiing, and fencing [11, 19, 20, 21]. Similar relationships between 2D:4D and success have been observed in completely different settings: low 2D:4D predicts success among high frequency financial traders [22], increased academic performance in male dentistry students [23] as well as better performance on a cognitive reflection task [24] and a Java programming course [25].

These findings, linking 2D:4D to (success in) competitive activities do at least suggest that 2D:4D may serve as a biomarker for dominance in humans as well. But evidence for a direct relationship between 2D:4D and dominance is mixed. Some studies do find a significant relationship with personality measures of dispositional [9], and aggressive dominance [26] but a similar number of studies does not observe any relationship [27, 28]. Interestingly, Manning and colleagues [9] ran a large scale study to assess the relationship between a dominance measure and 2D:4D among more than 150.000 participants, and found a correlation of *r* = -.03 between the dominance measure and both male and female 2D:4D. This relationship is weak, even after taking into account that due to the higher level of error in self-measured 2D:4D the actual correlation would be about three times higher [29]. Summarized, the literature illustrates a remarkable paradox: whereas 2D:4D is predictive for performance in sports, financial markets, and cognitive tasks that suggest the quest for dominance, there is no strong support for a direct linear relationship between 2D:4D and personality measures of dominance.

To unravel the paradox it is instructive to look, as other authors have done, at how context effects may modulate the relation between 2D:4D and behavioral outcomes [18, 30]. For instance, impulsiveness (as measured by financial discounting) appears only negatively related to male 2D:4D after a (experimentally manipulated) defeat in a competitive setting, but not after victory [31]. To the extent that impulsiveness is suboptimal behavior in the long run, this finding is at least consistent with the idea that low 2D:4D men are more dominant and therefore may have more difficulties in accepting this particular situation. Similarly, it has been shown that the relationship between 2D:4D and aggression is modulated by the setting in which the relationship is measured. Low 2D:4D predicts aggressive behavior only after watching a violent video [32, 33] and only predicts retributional responding following provocatively unfair offers [34]. These findings fit very well with findings on the relation between circulating testosterone levels and dominance-seeking behavior. In humans, measurements of testosterone, in blood or saliva, strongly predict status seeking behavior when an individual’s status is threatened, but not in the absence of a threat [35, 36]. In non-human primates testosterone relates to dominant and aggressive behavior only when the status hierarchy is unstable and dominance battles are possible [37, 38]. It has been proposed that testosterone only predicts dominant behavior when circumstances activate our “dominance” system, which is comprised of motivational and attentional processes that prepare us to take actions that allow us to maintain or enhance our social status [39]. Consistent with this perspective, particular facial cues are able to activate dominant responses in high testosterone individuals: after testosterone administration, eye contact—which may be seen as a display of dominance—is maintained longer when confronted with angry faces than with happy faces [40], even when these faces are masked. Given the observation that facial cues seem to be sufficient to activate a dominance related behavioral repertoire, we focus in the present study on the impact of facial characteristics on the relation between 2D:4D and dominance.

Facial cues have been shown to be important predictors for a wide range of social decisions, such as whether to trust an interaction partner in a trust game, whether to convict those who stand trial, or whether to vote for a specific candidate in an election [41, 42, 43]. These outcomes are at least partially driven by the fact that people promptly and unreflectively draw inferences about others’ intentions and personal dispositions on the basis of facial information and subsequently act upon these inferences [44]. In the case of the facial emotional expression of anger, the social signal is quite clear: the sender clearly communicates to the interaction partner the intention of a dominance clash. Such a social challenge seems to provoke retaliation in dominant individuals, while submissive and anxious individuals are more likely to give in [45]. Furthermore, these findings suggest that mere facial emotional expressions are able to activate one’s own dominance system and by doing so influence actions.

Interestingly, the dominance system also might be activated by facial cues irrespective of emotional content. In fact, Oosterhof & Todorov [46] identified trustworthiness and dominance as two dimensions that are sufficient to account for more than 80% of the variance in holistic neutral face evaluation. Research regarding specific stable morphological characteristics in emotionally neutral faces, especially those relating to facial maturity and masculinity, provides additional evidence that these cues trigger inferences related to dominance hierarchies and influence evaluations about not only physical strength [47], but also personal dispositions in relation to dominance and aggression: Variation, for instance, in “baby facedness”, characterized by stable morphological facial cues such as round faces and big eyes, influences perceptions of fitness and submissiveness [48]. Variation in facial width-to-height ratio in emotionally neutral faces predicts estimated propensity for aggression in others [49, 50]. More direct evidence that dominance-related facial cues activate the dominance system comes from neuroscientific findings that these cues preconsciously activate neural systems related to status and aggression [51, 52].

Considering that exposure to dominant-related facial cues can activate the dominance system and the observation that testosterone only predicts dominant behavior when circumstances “activate the dominance apparatus” [39], we propose that exposure to dominant facial characteristics enables the relation between 2D:4D and dominance to emerge. Specifically, we hypothesize that 2D:4D is more strongly related to dominance after exposure to dominant than to neutral or submissive facial cues.

## Method

Hundred-thirty-one heterosexual male participants between 18 and 38 years of age (90,8% (n = 119) Caucasian, 3.1% (n = 4) Chinese, 6.1% (n = 8) non-Chinese Asian) participated in this experimental study, which was approved by the Social and Societal Ethics Committee of the KU Leuven, and provided informed consent in writing. After reading the instructions participants completed the Social Value Orientation Slider Measure [53]. In this resource allocation paradigm, participants were asked 6 times how they would distribute monetary resources between themselves and another anonymous person. The responses to the six items, differing in joint payoff options, allow for the calculation of a person’s social value orientation. While the original paradigm does not include any information about the anonymous interaction partner, we slightly adapted the paradigm to allow for a between-subjects manipulation of facial dominance: when introducing the task to participants, we displayed a facial image of this hypothetical interaction partner, and we also showed this face before the six resource allocation decisions between the participant and the interaction partner. Participants were randomly allocated to a condition in which they were shown an emotionally neutral face with either submissive, neutral or dominant characteristics and reassured that mutual anonymity would be guaranteed. We manipulated the facial characteristics by using faces generated by a computer model that allows manipulation of facial dominance [54]. Oosterhof & Todorov’s extensively validated model [46] provides assurance that stimuli are well standardized, and specifically attuned to parametrically manipulate the facial dominance cues and associated trait dimension required for this research [46, 51, 54, 55]. The faces from this database were originally created using FaceGen 3.2 software [56]. We used two facial identities from Todorov’s dominance database [57] to enable generalization of the results. These were randomly allocated to participants. Three levels of dominance were chosen (-3, 0 and +3 SD on a normally distributed dimension) for each facial identity to determine submissive, neutral or dominant faces (see S1 Fig). This specific procedure allows to manipulate facial dominance characteristics while keeping other visual features of the interaction partner constant. As a result any difference in response can only be due to these changes in specific facial characteristics. Further, although imaginary, this particular setting and the decisions involved in this paradigm are assumed to come as close as possible to a real setting while keeping all other potential confounding factors controlled. Immediately after this task, participants were asked to complete the 11-item dominance scale of the International Personality Item Pool [58], a scale that focuses strongly on self-aggrandizing aspects of dominance and contains items like “I impose my will on others” and “I try to outdo others”. Participants responded to each item on a seven point scale from disagree (1) to agree (7). Finally, participants’ right hand was scanned to measure finger lengths. Participants placed their hand palm on the glass plate of a scanner and we ensured that details of major creases could be seen on the scans. Finger lengths were measured by two independent raters from the ventral proximal crease to the tip of the finger by means of the freeware program Autometric developed by DeBruine [59]. Finger lengths were measured from the bottom crease when there was a band of creases at the base of the digit [60].

## Results

Prior to the main analyses we calculated the Intraclass Correlation coefficient (ICC = .985), averaged the highly correlated 2D:4D measurements of both raters and used this average in our statistical analysis. We also tested the reliability of the dominance scale. Two items (“Hate to seem pushy” and “Demand explanations from others”) showed problematic factor loadings (< .30) in a PCA and were omitted from further analyses. The revised 9-item dominance scale resulted in a Cronbach’s Alpha of .74. After averaging the items of the dominance measure, we conducted a general linear model (GLM) analysis with facial dominance condition (collapsed across facial identities), 2D:4D (mean-centered) and the interaction between both as independent variables and with the averaged score on the International Personality Pool dominance scale as dependent measure. As predicted, we observed a significant interaction effect between 2D:4D and facial dominance condition, *F* (2, 125) = 3.50, *p* = .033, *η* ^2^ = .053, indicating that 2D:4D is negatively related to dominance in the dominant face condition (*r* = -.37, *p* = .01), but not in the neutral (*r* = .09, *p* = .58) or submissive (*r* = .06, *p* = .68) face conditions (see S2 Fig). No other effects turned out to be significant (all F’s < 2.2 and p’s > .12). Average age across conditions was comparable across conditions (21.8 years, 21.0 years and 21.1 years in the dominant, neutral and submissive conditions respectively), as was the average 2D:4D score (0.953, 0.956, 0.953, respectively). Prior research showed that Blacks and Chinese have lower 2D:4D than Caucasians [61]. In this study no Blacks participated, and only 4 participants were Chinese (3 in the dominant, none in the neutral and 1 in the submissive condition). Without the Chinese participants, the interaction between 2D:4D and facial dominance condition remained significant (F(2, 121) = 3.87, p = .023, *η* ^2^ = .060). The results are also robust across participant age. When age was added as a covariate, the interaction remained significant (F(2,124) = 3.48 *p* = .034, *η* ^2^ = .053, while the main effect of age was insignificant (F(1,124) = 0.013, p = .91, *η* ^2^ = .00).

## Discussion

Our findings provide new insights in different domains. First, our pattern of results sheds some light on earlier inconsistent findings with regard to whether 2D:4D can predict self-rated dominance. 2D:4D does reliably relate to actual and perceived facial features typically considered male and dominant [62, 63, 64], which implies the potential to activate the dominance system in interaction partners. Behavioral findings in both animal and human research also suggest that 2D:4D may serve as a biomarker of dominance [13–25]. Still, prior research only observed weak relationships between 2D:4D and explicit dominance measures [9, 26, 27, 28]. Our results resolve the inconsistency by taking into account that activation of the dominance system through particular contextual cues may predict when relationships between 2D:4D and dominance related behavior actually emerge [30–34]. The contexts that have been described to activate the dominance system are perceived as challenges or provocations, such as experiences of competition with other men in areas like territory formation, dominance disputes, or mate acquisition and guarding, or even challenges to one’s honor or reputation [65]. These environmental contingencies seem to heighten the importance of one’s position in the status hierarchy and trigger men’s striving for dominance [35, 36]. In line with this literature, we provide evidence in our study that merely activating thoughts about a presumed interaction that involves the distribution of resources with a dominant (but not a neutral or submissive) looking man may lead to the emergence of the relation between 2D:4D and explicit dominance measures. The present pattern of results accentuates that ignoring context will hide the existence of stable–but situated—relationships between 2D:4D and other variables. Second, whereas previous research regarding facial characteristics mainly focused on how facial cues guided trait inferences about the interaction partner [48, 49, 50, 51], our experimental results demonstrate that stable morphological facial cues related to dominance are able to influence inferences regarding one’s own biologically rooted personality predispositions with respect to dominance striving. Future research should investigate how this translates to a range of ecological distributive behaviors, common–for example–in buyer-seller interactions or organizational settings. Another path for future research may focus on the mechanism behind the effect. Some evidence suggests that low 2D:4D relates to high testosterone production after aggressive and/or physical challenges [66, 67, 68]. Given that testosterone especially predicts dominance when the dominance system is activated [39] these circulating testosterone levels may explain why a relationship between 2D:4D and dominance especially emerges when men are exposed to dominant opponents.

Based on the findings in the current study, we suggest that across many domains emotionally neutral dominant facial cues will activate the dominance system and increase vigilance, that is, men may be prepared for competition when meeting a dominant other. This vigilance may be captured in the relation we observed between 2D:4D and dominance after hypothetical interaction with a dominant other. This does not mean that mere interaction with a dominant other always leads to competitive action, as dominant others do not necessarily have to fight for dominance. Indeed, if facial dominance cues represent an interaction partner’s capability to inflict harm, rather than their intent [46], mental preparation might be an adaptive response until other cues clarify which action is appropriate. In view of the literature regarding the ‘challenge hypothesis’ [35, 36, 65], an interaction partner’s capability to inflict harm might just alert a man to a potential challenge or provocation, and hence merely lead to a state of mental preparedness. In line with findings relating to responses to angry faces [45], a potential challenge by a dominant looking individual seems to provoke mental preparation for dominance-striving in individuals with a biologically dominant nature, while individuals with a biologically submissive nature are more likely to mentally prepare for yielding or withdrawal. Being mentally vigilant and prepared for a potential dominance clash is adaptive for dominant men as it allows them to strategically optimize their behavior to increase their chances to maintain or enhance status. It is equally adaptive for submissive men as it potentially allows them to respond in a way that avoids the interaction to evolve into a harmful one. As Louis Pasteur once said: ‘Chance favors the prepared mind’; it might just be that men in the face of latent conflict with substantial costs and benefits mentally prepare for the appropriate response in order to optimize their chances of success.

## Conclusions

The present study provides experimental evidence that male 2D:4D is related to a dominance measure after (fictitious) male-male interaction when the other man has a dominant, but not a submissive or neutral face. This finding provides evidence for the idea that the interaction with a dominant other activates the dominance system which leads to the emergence of a negative correlation between 2D:4D and dominance.

## Supporting Information

S1 Fig Facial dominance manipulation. (TIF)

S2 Fig Relationship between right hand 2D4D and dominance by facial condition. (TIF)

S1 File The supporting dataset file. (SAV)
