# Supplementary figures and images for: The Influence of Facial Characteristics on the Relation between Male 2D:4D and Dominance
Source: PLoS One. 2015 Nov 23;10(11):e0143307. doi: 10.1371/journal.pone.0143307 (PMC4657903; doi:10.1371/journal.pone.0143307)

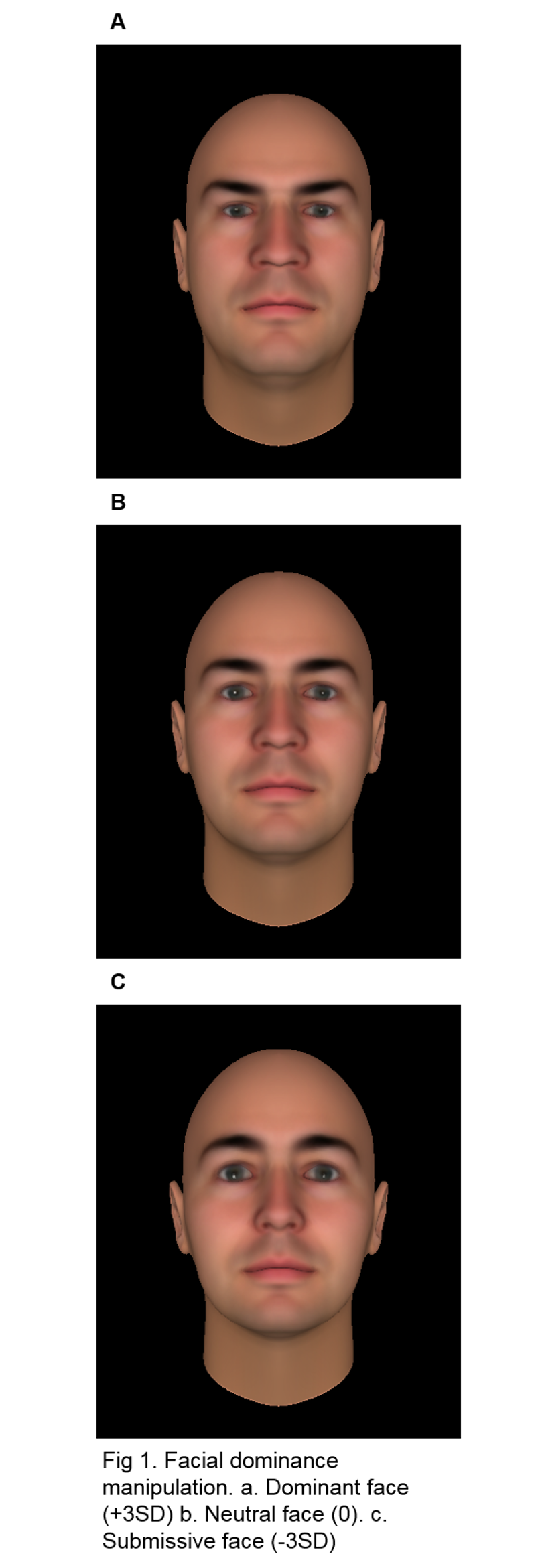

Supplement: S1 Fig — (TIF) [file pone.0143307.s001.tif]

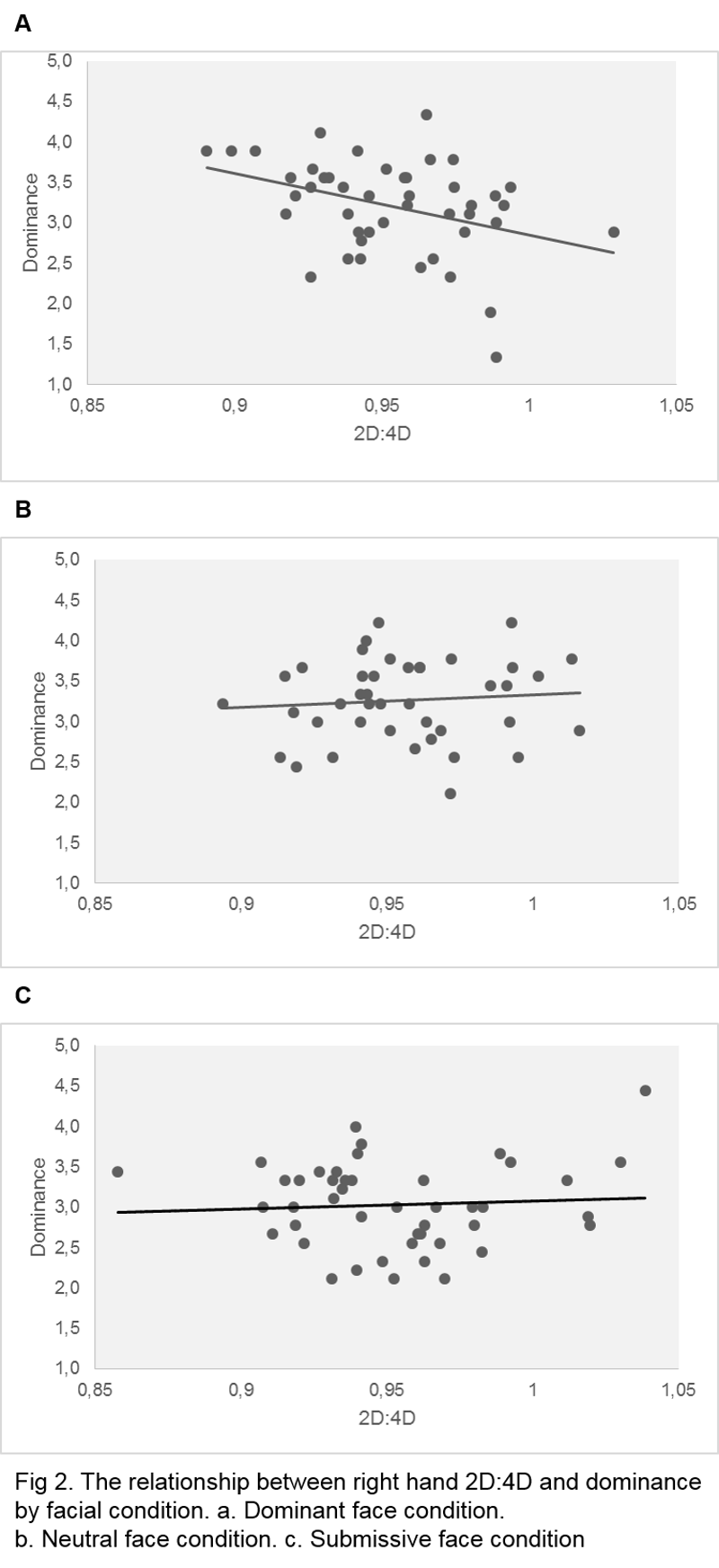

Supplement: S2 Fig — (TIF) [file pone.0143307.s002.tif]
